# Adventitious rooting in response to long-term cold: a possible mechanism of clonal growth in alpine perennials

**DOI:** 10.3389/fpls.2024.1352830

**Published:** 2024-04-17

**Authors:** Priyanka Mishra, Adrian Roggen, Karin Ljung, Maria C. Albani, Alice Vayssières

**Affiliations:** ^1^ Institute for Plant Sciences, University of Cologne, Cologne, Germany; ^2^ Cluster of Excellence on Plant Sciences, “SMART Plants for Tomorrow’s Needs,” Heinrich-Heine University Düsseldorf, Düsseldorf, Germany; ^3^ Max Planck Institute for Plant Breeding Research, Cologne, Germany; ^4^ Department of Botany, Faculty of Science, University of Allahabad, Prayagraj, India; ^5^ Umeå Plant Science Centre, Department of Forest Genetics and Plant Physiology, Swedish University of Agricultural Sciences, Umeå, Sweden; ^6^ Rijk Zwaan, De Lier, Netherlands; ^7^ Université Paris-Saclay, INRAE, AgroParisTech, Institut Jean-Pierre Bourgin (IJPB), Versailles, France

**Keywords:** adventitious root, alpine, *Arabis alpina*, clonal propagation, extended cold exposure, phytohormones, transcriptome

## Abstract

Arctic alpine species experience extended periods of cold and unpredictable conditions during flowering. Thus, often, alpine plants use both sexual and asexual means of reproduction to maximize fitness and ensure reproductive success. We used the arctic alpine perennial *Arabis alpina* to explore the role of prolonged cold exposure on adventitious rooting. We exposed plants to 4°C for different durations and scored the presence of adventitious roots on the main stem and axillary branches. Our physiological studies demonstrated the presence of adventitious roots after 21 weeks at 4°C saturating the effect of cold on this process. Notably, adventitious roots on the main stem developing in specific internodes allowed us to identify the gene regulatory network involved in the formation of adventitious roots in cold using transcriptomics. These data and histological studies indicated that adventitious roots in *A. alpina* stems initiate during cold exposure and emerge after plants experience growth promoting conditions. While the initiation of adventitious root was not associated with changes of *DR5* auxin response and free endogenous auxin level in the stems, the emergence of the adventitious root primordia was. Using the transcriptomic data, we discerned the sequential hormone responses occurring in various stages of adventitious root formation and identified supplementary pathways putatively involved in adventitious root emergence, such as glucosinolate metabolism. Together, our results highlight the role of low temperature during clonal growth in alpine plants and provide insights on the molecular mechanisms involved at distinct stages of adventitious rooting.

## 1 Introduction

Adventitious roots develop post-embryonically on non-root tissues and are important to support plants growing in unstable substrate and enabling accidental or active clonal growth through creeping stems (Körner, 2003). Adventitious rooting is regularly used to multiply several horticultural species through cuttings (de Klerk et al., 1999; Steffens and Rasmussen, 2016). The process of adventitious rooting is divided into four successive stages, cell dedifferentiation followed by root meristem induction, initiation, and emergence (Kevers et al., 1997; Klerk and De Klerk, 2002). In the first stage, cells dedifferentiate to reacquire cell proliferation potential and are established as the founder cells competent to respond to inductive signals (Klerk and De Klerk, 2002). These cells have been observed to originate in response to different stimuli and from different cell types that vary between species (Geiss et al., 2009). Afterwards, the formation of a root meristem is induced, and the root meristem is initiated starting with division of the founder cells (Kevers et al., 1997; Klerk and De Klerk, 2002). In the final phase, the root primordium develops and emerges out of the stem. The regulation of adventitious rooting is very complex and involves the interaction of several phytohormones with auxin, and specifically the maintenance of an auxin gradient being crucial in this process (Lakehal and Bellini, 2018). Phytohormones, such as brassinosteroid, cytokinin, ethylene, gibberellic acid and jasmonic acid regulate auxin metabolism and signaling during adventitious root initiation. Among these phytohormones, some (auxin, brassinosteroids and ethylene) function as positive regulators, whereas others (abscisic acid, cytokinin, gibberellic acid, jasmonic acid and strigolactone) act as negative regulators during the process of adventitious rooting.

In nature, clonal growth is a typical characteristic of perennial plants. The formation of adventitious roots from non-root tissues can be induced during normal development (nodal roots in strawberry) and in response to stress conditions such as wounding, herbivory, nutrient deprivation, and flooding by modulating a complex cascade of different phytohormones (Bellini et al., 2014). In response to flooding, for example, ethylene biosynthesis increases leading to adventitious root primordia formation following a complex interplay of ethylene with auxin, gibberellin, abscisic acid, and reactive oxygen species (Sauter, 2013; Voesenek and Bailey-Serres, 2015; Steffens and Rasmussen, 2016). Climatic conditions, specifically long-term exposure to low temperature, as factors influencing adventitious root formation have not been extensively addressed. Alpine perennials have developed the ability to propagate clonally in a bid to increase fitness during unfavorable conditions like longer winters that hamper successful flowering and seed set (Billings and Mooney, 1968). The tendency to propagate vegetatively has also been hypothesized to positively correlate with increasing elevation in conjunction with decrease in temperature (Billings and Mooney, 1968; Bliss, 1971). Cold treatment on cuttings is practiced in horticultural protocols to improve the rooting efficiency in difficult-to-root species (Diaz et al., 1987). In cuttings, exposure to cold has been reported to promote and, in some cases, inhibit the formation of adventitious roots (Brix, 1974; Gesto et al., 1981; Diaz et al., 1987; Garrido et al., 1996, 1998; Kibbler et al., 2004; Brondani et al., 2012; Jeon and Kim, 2013; De Almeida et al., 2017).

In this study, we explored the effect of the duration of long-term cold exposure on adventitious rooting in *Arabis alpina*, an arctic-alpine Brassicaceae species that has been used as a model to study the regulation of perennial traits within the Brassicaceae family (Ansell et al., 2008; Wang et al., 2009; Tedder et al., 2011; Karl et al., 2012; Bergonzi et al., 2013; Lazaro et al., 2018; Hughes et al., 2019; Vayssières et al., 2020)*. A. alpina* is found in habitats showing broad ecological niches in terms of latitude, elevation and soil types distributed among the European Alps, Spain, Northern and Eastern Africa, Asia Minor, Scandinavia, and parts of North America (Tedder et al., 2011; Laenen et al., 2018). *A. alpina* has been previously reported to produce adventitious roots on stems of intact plants and to show natural variation in adventitious rooting (Mishra et al., 2020). However, this study focused on accessions that do not require cold exposure to flower. Most alpine plants have acquired, in addition to clonal propagation, additional traits such as seed dormancy, inflorescence preformation, bud dormancy, and vernalization requirement (Billings and Mooney, 1968; Körner, 2003; Rohde and Bhalerao, 2007; Fernández-Pascual et al., 2021). To explore how an *A. alpina* accession that requires vernalization to flower forms adventitious roots and how these traits could be linked, we focused our studies on the *A. alpina* accession Pajares, an obligate vernalization-dependent ecotype (Wang et al., 2009; Albani et al., 2012). Pajares has a fully sequenced genome and has been characterized for its flowering behavior and shoot architecture in response to different durations of cold exposure (Willing et al., 2015; Lazaro et al., 2018; Vayssières et al., 2020). For flowering, the length of the cold period positively associates with the inflorescence outgrowth (Lazaro et al., 2018). In this study, we showed that prolonged cold exposure was also positively associated with the presence of adventitious roots at specific internodes of flowering *A. alpina* plants. We demonstrated that adventitious root primordia at these internodes are induced, initiated, and emerged during cold. Taking advantage of this spatial pattern of adventitious rooting in flowering plants, we analyzed the transcriptome of stems after different durations of cold treatment and identified genes that may be involved at distinct stages of adventitious rooting in response to cold.

## 2 Materials and methods

### 2.1 Plant materials, growth conditions and phenotyping

**Figure 1:**
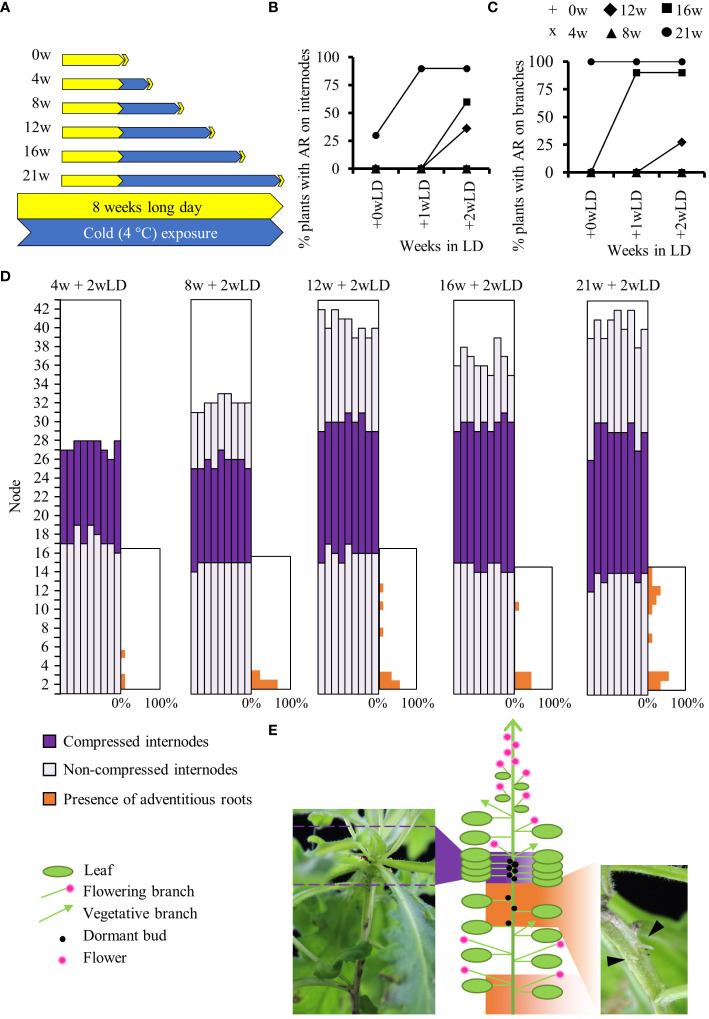
Physiological analysis of adventitious rooting in *A. alpina* in response to different durations of cold exposure. **(A)** Diagrammatic representation of the experimental design. *A. alpina* plants were grown for eight weeks in a LD greenhouse (0w), were subjected to 4°C for 4 (4w), 8 (8w), 12 (12w), 16 (16w) and 21 (21w) weeks and subsequently were transferred back to a LD greenhouse. Yellow represents LD greenhouse conditions and blue a 4°C LD growth chamber condition. Percentage of Pajares plants with adventitious roots on the main stem **(B)** and the axillary branches **(C)** after exposure to different durations (0w, 4w, 8w, 12w, 16w and 21w) at 4°C followed by 0, 1, and 2 weeks in long days (+wLD). **(D)** Analysis of the position of adventitious root formation along the main stem in a set of *A. alpina* plants after exposure to different durations (4w, 8w, 12w, 16w and 21w) at 4°C followed by 2 weeks in long days (+2wLD). Each column represents a single plant. The percentage of adventitious root in each internode along the main stem is indicated in orange on the right side of each graph. Internodes are numbered from the bottom to the top of the plant. **(E)** Schematic representation of a 21w flowering *A. alpina* plant, in the right inset a picture of a stem in the rooting internodes (21w + 2w) with adventitious roots (arrows), in the left inset a picture illustrating the compressed internodes zone (8w + 0w). Shoot architecture of a flowering *A. alpina* plant consists of zones of flowering axillary branches, dormant buds, and vegetative axillary branches. In **(D)** and **(E)**, dark purple represents compressed internodes, light purple extended internodes and orange represents internodes with adventitious roots. n > 10 in **(B)** and **(C)** and n = 9 in **(D)**.

For physiological experiments, we used the *A. alpina* accession Pajares and *pDR5::GUS* Pajares lines previously described in Vayssières et al., 2020. Pajares was collected in the Coedillera Cantábrica mountains in Spain at 1,400 m altitude (42°59′32′′ N, 5°45′32′′ W) (Wang et al., 2009). Vernalization experiments were performed as described before (Lazaro et al., 2018; Vayssières et al., 2020; Viñegra de la Torre et al., 2022) except that a constant photoperiod (LD, 16h light: 8h dark) was maintained throughout the experiment to specifically study the effect of long-term cold on adventitious rooting. Hence, the plants were grown in soil in a glasshouse under long-day conditions (LD; 16 h light: 8 h dark) at 20°C/18°C day/night temperatures until they were 8 weeks old, and were then moved to growth chambers maintained at 4°C and LD conditions for 4, 8, 12, 16 and 21 weeks (**Figure 1A**). After cold treatment, plants were moved back to the LD glasshouse conditions (**Figure 1A**). The presence of adventitious roots on internodes of the main stem and on axillary branches of each plant was scored before cold treatment, at the end of each cold treatment, 0 week in LD (+0wLD) and after plants were returned to glasshouse conditions for 1 (+1wLD) and 2 (+2wLD) weeks. The presence of adventitious roots was also monitored on 8 weeks old plants maintained in LD condition for 5 additional weeks (+5wLD) (**Supplementary Figure 1C**). These plants had the same number of internodes as the flowering plants being exposed to 21 weeks in cold and returned to glasshouse conditions for 2 weeks (21w + 2wLD). Only the presence and not the number of adventitious roots on the internodes was scored (**Figure 1E**; picture on the right). The percentage of plant with adventitious root on the internodes or on branches was calculated. Due to the unique spatial presence of adventitious roots on the main stem we decided to characterized it further using the percentage of the presence of adventitious roots per internode position along the main stem. The internodes of the main stem that develop during cold exposure did not elongate and were refer to as “compressed internode zone” compared to the internodes that develop before and after cold exposure that showed elongation and were refer to as “non-compressed internode zone” (**Figure 1E**; picture on the left). We scored the percentage of flowering plants and the total number of leaves at flowering in 8 weeks old plants exposed to different durations (0w, 4w, 8w, 12w, 16w and 21w) at 4°C followed by 4 weeks in long days (+4wLD). At least 9 plants were used for each condition.

### 2.2 Library preparation and RNA sequencing and analysis

For the transcriptome analysis, we harvested internodes 9 – 13, that show specific adventitious root formation in plants after long term cold treatment. Around 2 cm stem section excluding the axillary buds was collected from Pajares plants before cold exposure (0w), after being exposed for 4, 8, 12, 16 and 21 weeks to 4°C (4, 8, 12, 16 and 21w; +0wLD) and after being exposed to 12, 16 and 21 weeks to 4°C and transferred back to a LD glasshouse for 5 days (12, 16 and 21w; +5dLD) (**Figure 1A**). This stem section corresponded to the 5 internodes below the compressed internode zone. Given that plants cultivated in long-day conditions without cold exposure (8wLD + 5LD) did not flower, therefore did not have the same shoot architecture (lacking the compressed zone) and did not share the same age, we didn’t include them in the transcriptomic analysis. Each sample consisted of stems from 10 plants and harvested in three biological replicates. The purified RNA samples were sent to the Max Planck Genome Center, Cologne, Germany, for library preparation and sequencing (https://mpgc.mpipz.mpg.de/home/). 1 µg of total RNA was enriched for poly-A RNA using the NEBNext Poly(A) mRNA Magnetic Isolation Module (New England Biolabs), followed by library preparation using the NEBNext Ultra Directional II RNA Library Prep kit (New England Biolabs). RNA quality and quantity were monitored throughout by capillary electrophoresis (TapeStation, Agilent Technologies) and fluorometry (Qubit, Thermo Fisher Scientific). Sequencing was performed on a HiSeq3000 device (Illumina) generating 150-bp single-end reads. Approximately 12.5 million reads were sequenced from each library. Before further processing, the sequencing data quality was verified using FastQC (https://www.bioinformatics.babraham.ac.uk/projects/fastqc/). Sequences were mapped and aligned to the reference genome using HISAT2 followed by assembly and quantification of expression levels in different samples using STRINGTIE (Pertea et al., 2016). The gene counts of all samples were obtained using a Python script (http://ccb.jhu.edu/software/stringtie/dl/prepDE.py). The quality of the samples was assessed by producing dispersion plots among replicates. The differentially expressed genes with more than 2-fold change and a corrected p-value below 0.05 were obtained using DESeq2 and selected for further analysis. Transcriptome data from this study is deposited in the Gene Expression Omnibus database (https://www.ncbi.nlm.nih.gov/geo/) under the accession number GSE176054. For Gene Ontology (GO) term enrichment, the *R*-based graphical tool BACA (Bubble chArt to Compare Annotations) generated using DAVID was used to visualize (Dennis et al., 2003; Fortino et al., 2015). A bubble chart was generated summarizing the enriched GO terms (Benjamini corrected p-value < 0.05) among the modulated genes in different samples. The size of each bubble representing a GO term denotes the number of genes in a list of differentially expressed genes associated with the GO term, and the color indicates the direction of modulation (green = downregulation, magenta = upregulation). A minimum of five genes was required for the GO term to be considered for further analysis. The enrichment index of hormone responsive genes was calculated in the following manner: Enrichment = (number of up- or down-regulated genes responding to a hormone/number of all genes responding to that hormone)/(number of all up- or down-regulated genes/number of all genes). We assumed in this case that the *A. alpina* orthologues respond in an analogous manner. The hormone responsive genes correspond to the respective GO terms collected from AmiGO. The following GO terms were considered: response to abscisic acid (GO:0009737), auxin (GO:0009733), brassinosteroid (GO:0009741), cytokinin (GO:0009735), ethylene (GO:0009723), gibberellin (GO:0009739), jasmonic acid (GO:0009753) and salicylic acid (GO:0009751) (**Supplementary Dataset 1**). The significance of the enrichment was estimated using Monte Carlo method by testing whether the overlap between the sets of hormone responsive genes and the differentially regulated genes was higher than it would be expected by chance. The test consists of randomly selecting a number of differentially regulated genes from the *A. alpina* genome 10,000 times. We thus counted the number (n) of times that the intersection size of the random lists was equal to or higher than the intersection observed for two lists of tested genes. A *P value* of n/10,000 was then calculated. Strigolactone responsive gene enrichment was not studied due to the small number of gene in the GO terms (7 genes). Genes sharing similar expression profiles were identified using Cluster 3.0 (Eisen et al., 1998) using the same parameters as in Vayssières et al., 2020. Genes were selected out of the total of 30510 genes based on the following criteria: log_2_(Fold-change) ≥ 2 and the difference between the maximum and the minimum value ≥ 3. Heatmaps were generated for the genes passing this threshold using TreeView based on the values obtained from Cluster 3.0 (de Hoon et al., 2004). A group of genes showing similar distribution in expression pattern were grouped into a cluster. The genes and corresponding GO terms in the clusters representing the distinct expression profiles were further analyzed.

### 2.3 GUS staining

For GUS staining assays, stems from the last extended internodes below the compressed internode zone were harvested from *pDR5::GUS A. alpina* plants grown for 8 weeks in a LD greenhouse, and after being exposed to 4 °C for 4 weeks and 21 weeks. After harvesting, stem samples were incubated for 1 h in 90% ice-cold acetone and were subsequently washed in 50 mM phosphate buffer (pH 7.0) and submerged in 2.5 mL GUS staining solution under moderate vacuum for 20 min. The GUS staining solution was composed of 1 mM EDTA (pH 8), 5 mM Potassium ferricyanide, 5mM Potassium ferrocyanide, 100 mM Sodium phosphate (pH 7.0), 1% Triton-X-100, 1 mg/mL X-Gluc (Cold Spring Harb Protoc, 2007; https://doi.org/10.1101/pdb.rec10860). Samples were incubated afterwards at 37°C in the dark for a maximum of 16 h and subjected to ethanol washes to remove the chlorophyll. Transverse sections of 70 µm thickness were prepared using a Leica VT1000S vibratome from samples immobilized on 6% (w/v) agarose. Representative photographs from two biological replicates were obtained using the Zeiss Axio Imager microscope with the Zeiss Axio Cam 105 color camera (Zeiss, Jena, Germany) or the Nikon Eclipse Ni-U microscope with the Nikon Digital Sight camera (DS-Fi2) (Nikon, Tokyo, Japan).

### 2.4 Free endogenous IAA quantification

The three biological replicates of stem material harvested for the transcriptome analysis were also used for endogenous free IAA quantification. Around 15 mg of plant material (fresh weight) was processed as previously described (Andersen et al., 2008). Free IAA was quantified in the purified samples using combined gas chromatography - tandem mass spectrometry. 500 pg ^13^C_6_-IAA internal standard was added to each sample before homogenization and extraction. Values represent the average of three biological replicates and error bars represent the standard deviation of the mean.

### 2.5 Gene expression analysis

For the quantification of *GUS* expression in *pDR5::GUS* plants, stem samples were collected following the protocol used for sample collection for the transcriptome analysis. Total RNA was extracted from the samples using the RNeasy Plant Mini kit (QIAGEN) following the manufacturer’s protocol. Residual DNA was removed using the DNA-free DNA removal kit (Invitrogen). The RNA concentration and integrity were determined using a NanoDrop ND-1000 UV-Vis spectrophotometer (Thermo Fisher Scientific). Total RNA (2 µg) was used for the synthesis of cDNA using SuperScript II Reverse Transcriptase (Invitrogen) and oligo dT (18) primers. 3 μL of each cDNA solution (1:5.5 dilution) was used as a template for each quantitative PCR reaction (qPCR) performed in three technical replicates. The CFX Connect Real-Time PCR Detection System (Bio-Rad) and the iQ SYBR Green Supermix (Bio-Rad) were used to perform the real-time quantitative PCR analyses using the primers: GUS-F – CCCTTACGCTGAAGAGATGC, GUS-R – TTCGTTGGCAATACTCCACA, AaPP2A-F – AGTATCGCTTCTCGCTCCAG and AaPP2A-R - AACCGGGTTGGTCGACTATTG. The PCR programme followed was 95°C for 3 min, followed by 40 cycles of 95°C for 15 s and 60°C for 60 s, then a melting curve (55–95°C with a heating rate of 0.5°C/5 s). Values represent the average of three biological replicates and error bars represent the standard deviation of the mean.

## 3 Results

### 3.1 Prolonged cold exposure induces adventitious roots in *A. alpina* main stem and axillary branches

To test whether the length of cold exposure influences clonal growth through adventitious roots in *A. alpina*, we exposed plants of the Pajares accession to different durations of cold treatment (**Figure 1A**) (Wang et al., 2009). We observed that plants that did not experience cold treatment or experienced cold for only 4 and 8 weeks did not show adventitious roots on the main stem or in the branches (**Figures 1B, C**). Plants cold-treated for 21 weeks had a higher propensity to produce adventitious roots on the main stem and axillary branches compared to plants treated with shorter durations of cold (12 and 16 weeks) (**Figures 1B, C**). The length of cold exposure had a quantitative effect on adventitious rooting in the internodes of the main stem and in axillary branches. Also, the longer the plants were exposed to cold, the sooner we observed adventitious roots after plants were returned to greenhouse conditions (**Figures 1B, C**). Overall, these results suggest that long term cold exposure leads to adventitious root formation.

On the main stem, we observed adventitious roots developed preferentially on specific internodes (**Figures 1D, E**). Plants cold treated for 21 weeks produced adventitious roots on the lower internodes of the main stem (33.3% of plants on first internode and 55.6% on the second internode) and on the internodes 9 to 13. Specifically, 11.1% of plants produced adventitious roots on internodes 9, 12 and 13, 33.3% on internode 11 and 22.2% on internode 10, respectively (**Figure 1D**). Adventitious roots on lower internodes appeared also in non-cold treated plants and on plant cold treated for shorter time, whereas the ones at higher internodes were specific to plants after long term cold treatment (**Figure 1D** and **Supplementary Figure 1D**). We have previously shown that in greenhouse conditions, plants of the Pajares accession occasionally develop adventitious roots in the lower internodes but not in the upper internodes (Mishra et al., 2020). These results suggest that like in other species, adventitious roots in *A. alpina* at lower internodes develop in response to soil humidity (Stevenson and Laidlaw, 1985). Adventitious roots at higher internodes were mostly observed below a zone of compressed (short) internodes that contain dormant buds that shape the perennial shoot architecture of *A. alpina* (**Figure 1E**) (Vayssières et al., 2020). Interestingly, internodes 9 to 13 on the main stem were the last to expand before the plants were exposed to cold (purple in **Figure 1D**).

As previously noted in *A. alpina* accession Pajares, plant cold-treated for 4 weeks did not flower and flowered only partially for plant treated for 8 weeks (only 50% of the plant flowered; **Supplementary Figure 1A**) (Wang et al., 2009; Lazaro et al., 2018). Plants cold-treated for 12, 16 or 21 weeks flowered and showed the same total leaf number at flowering (**Supplementary Figures 1A, B**). We followed plants that haven’t been treated by cold until they reached the same number of leaves as the plants cold-treated for 12, 16 and 21 weeks (39.7 leaves). In these plants, no adventitious roots were observed on the main stem unlike as observed for the plants cold-treated for 12, 16 and 21 weeks. This result suggests that the total number of internodes does not influence adventitious rooting, but stem aging in cold does (**Supplementary Figures 1C–F**, **Figure 1D**). This also suggests an age- or position- dependent response to cold treatment for the development of adventitious roots on the main stem.

### 3.2 Transcriptome analysis of adventitious rooting in upper internodes reveals the enhancement of transcript accumulation of root-associated genes during cold exposure

**Figure 2:**
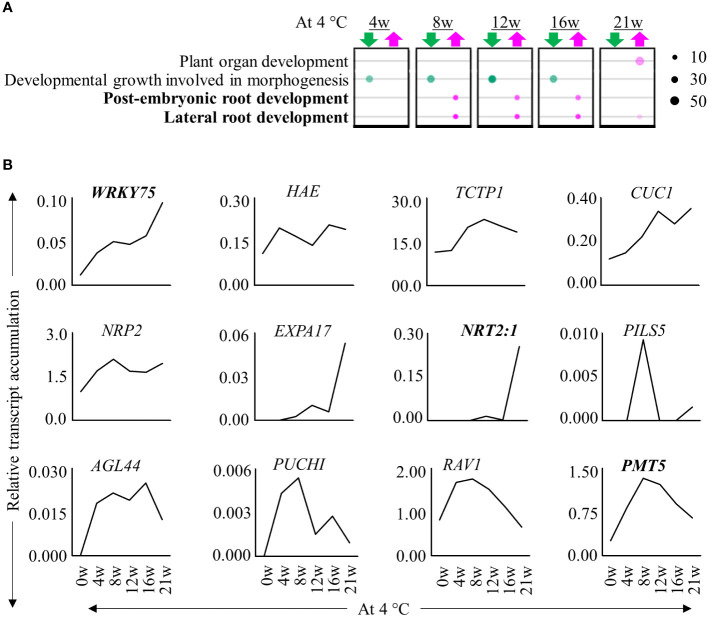
Expression patterns of root-associated genes during extended cold exposure. **(A)** Bubble chart showing selective enriched GO terms. The upregulated (magenta) and downregulated (green) genes in the rooting zone at the end of 4w, 8w, 12w, 16w and 21w of cold (4°C) exposure relative to before cold exposure were analysed. The size of the circle denotes the number of genes participating. The transparency of the circle represents the confidence with respect to the set Benjamini-corrected *p*-value of 0.01. The GO terms in bold are root associated. **(B)** The relative expression levels of homologs of root-associated genes, *AaWRKY75*, *AaHAE*, *AaTCPT1*, *AaCUC1*, *AaPUB9*, *AaNRP2*, *AaEXPA17*, *AaNRT2:1*, *AaPILS5*, *AaAGL44*, *AaRAV1* and *AaPMT5*, were differentially regulated throughout cold exposure until 21 weeks. Expression values are the average counts relative to the housekeeping gene, *AaPP2A*. Genes in bold have been described to be involved in adventitious root formation.

We performed a transcriptome profiling to understand how prolonged cold triggers the formation of adventitious roots at internodes 9 – 13. We compared stem samples of plants harvested before, during cold exposure for 4, 8, 12, 16 and 21 weeks and after they were transferred back to greenhouse conditions for 5 days (+5dLD) (**Figure 1A**). Of the differentially expressed genes (DEGs) we identified, 73.4% had homologues in *A. thaliana* (**Supplementary Table 1**; **Supplementary Figures 2A, B**). Comparisons of the transcriptome before (0w) and during cold exposure (4w/8w/12w/16w/21w) indicated that a higher number of DEGs were down- than upregulated during cold exposure (**Supplementary Table 1**; **Supplementary Figure 2A**). There was an overlap of DEGs in stem samples harvested during different durations of cold treatments (**Supplementary Figure 2A**). We then compared the transcriptome of stem samples at the end of cold treatment (+0wLD) with the transcriptome of stem samples after cold treatment (+5dLD) in plants exposed to 12w, 16w and 21w of cold. In these comparisons, we found that more genes were up- than downregulated after cold treatment (**Supplementary Figure 2B**). Expression profiles of samples during cold exposure clustered separately compared to the ones collected in LD greenhouse conditions both before and after cold treatment (**Supplementary Figure 2C**). Among the samples collected during cold, internodes from 21w cold treated plants clustered separately from the rest suggesting that the duration of cold exposure changes the transcriptome of the internodes that have a higher propensity to produce adventitious roots. We observed an enrichment of GO terms associated with lateral and post-embryonic root development in internodes harvested at the end of 8w, 12w, 16w and 21w of cold treatment compared to before treatment (0w) (**Figure 2A**). Accordingly, homologs of transcripts of root development-associated genes were upregulated in the internodes during cold treatment, few of which have been described to be involved in adventitious root development (**Figure 2B**) (Li et al., 2018; Tahir et al., 2021; Zhang et al., 2021). This includes *PUCHI*, *AGAMOUS-LIKE 44* (*AGL44*), HAESA (*HAE*), *Related to ABI3/VP1* (*RAV1*), *plant U-box 9* (*PUB9*) and *polyol/monosaccharide transporter 5* (*PMT5*), *CUP SHAPED COTYLEDON 1* (*CUC1*), *WRKY DNA-BINDING PROTEIN 75* (*WRKY75*), *EXPANSIN A17* (*EXPA17*), *Translationally Controlled Tumor Protein 1* (*TCTP1*), *NUCLEOSOME ASSEMBLY PROTEIN 2* (*NRP2*) and *NITRATE TRANSPORTER 2.1* (*NRT2:1*) (Hu et al., 2004; Remans et al., 2006; Zhu et al., 2006, 2019; Devaiah et al., 2007; Hirota et al., 2007; Lee and Kim, 2013; Deb et al., 2014; Li et al., 2018; Alvarez-Buylla et al., 2019; Branco and Masle, 2019). The increase in expression of root-associated genes suggest that adventitious root primordia develop on the internodes during cold exposure.

**Figure 3:**
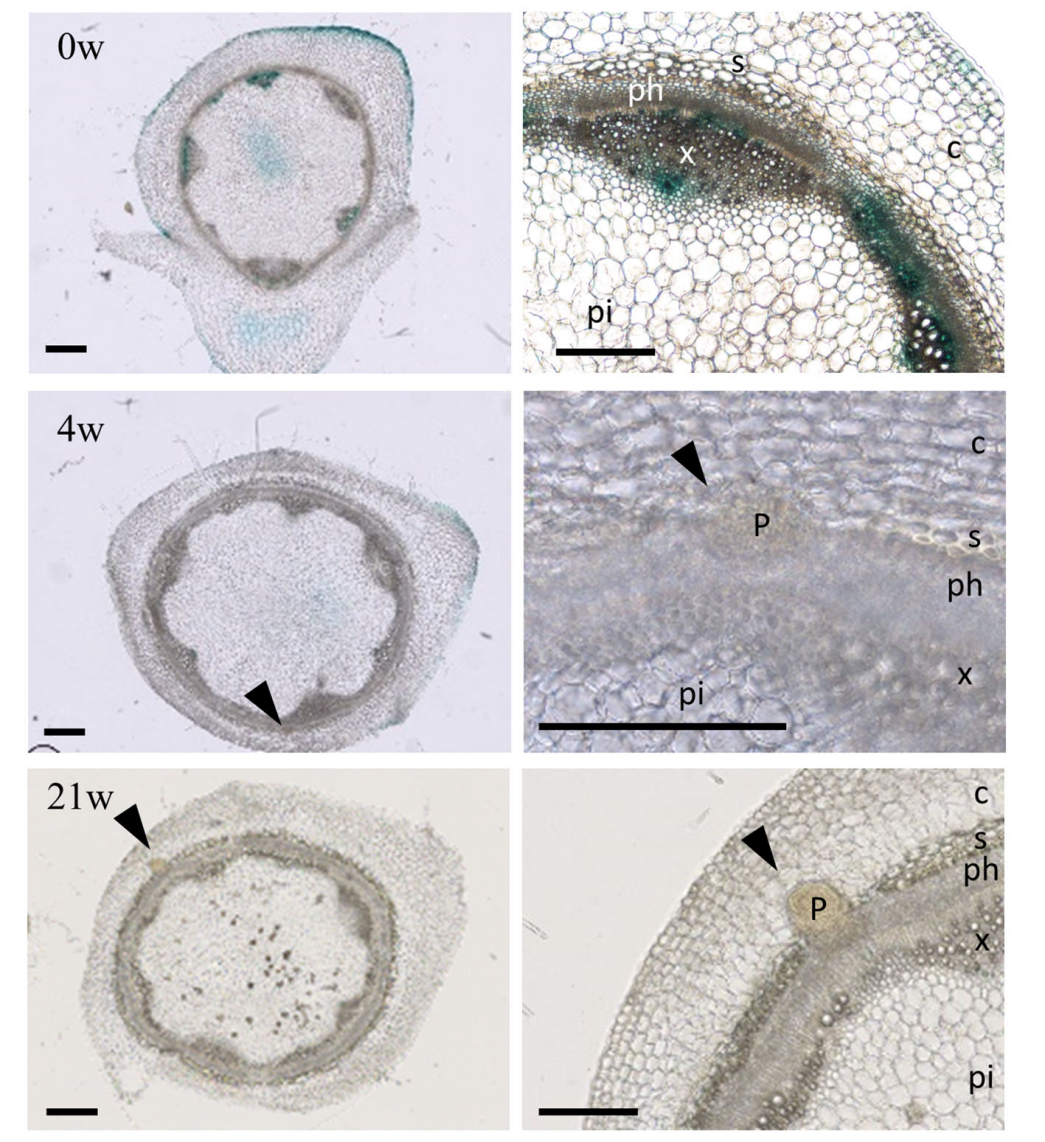
Adventitious root primordia on stems during extended cold exposure. Histological sections of internodes 9 - 13 from plants grown for eight weeks in LD (0w) and cold-exposed for 4 (4w) and 21 (21w) weeks. Arrow heads point adventitious root primordia (also shown in the insets). Bars, 500 µm. P, adventitious root primordia; c, cortex; s, sclerenchyma; x, xylem; ph, phloem; pi, pith.

To verify whether adventitious root primordia formation on the stems initiate during cold treatment, we used the *A. alpina* lines carrying the synthetic *DR5* promoter fused to the reporter gene *GUS* (**Figure 3**) (Vayssières et al., 2020). Reporter plants transformed with *pDR5::GUS* have been used in several species to monitor developing adventitious roots following auxin accumulation (Inukai et al., 2005; Sukumar et al., 2013; Rasmussen et al., 2015). In *A. alpina* stems, *GUS* expression under *pDR5* during cold exposure was reduced in comparison to before cold exposure suggesting a decreased *GUS* transcription in cold as described before (**Figure 3**; **Supplementary Figure 3**) (Lee et al., 2005). Nevertheless, stem sections in internodes 9-13 during different durations of cold treatment demonstrated the presence of adventitious root primordia during but not before cold treatment (**Figure 3**). In stem sections harvested from plants after 4 weeks of cold treatment, we observed the presence of a meristematic mass, whereas the ones harvested from plants cold treated for 21 weeks had established root primordia (**Figure 3**). Root primordia in *A. alpina* were present in the stem vasculature, at the border of xylem and phloem. These results suggest that adventitious roots in the main stem are formed in response to cold exposure and that prolonged duration of cold treatment may be important to ensure the development of adventitious roots. Emergence of adventitious root primordia may occur during or after the cold exposure.

### 3.3 Transcriptome analysis reveals co-expressed gene clusters across developmental stages of adventitious roots in *A. alpina*

**Figure 4:**
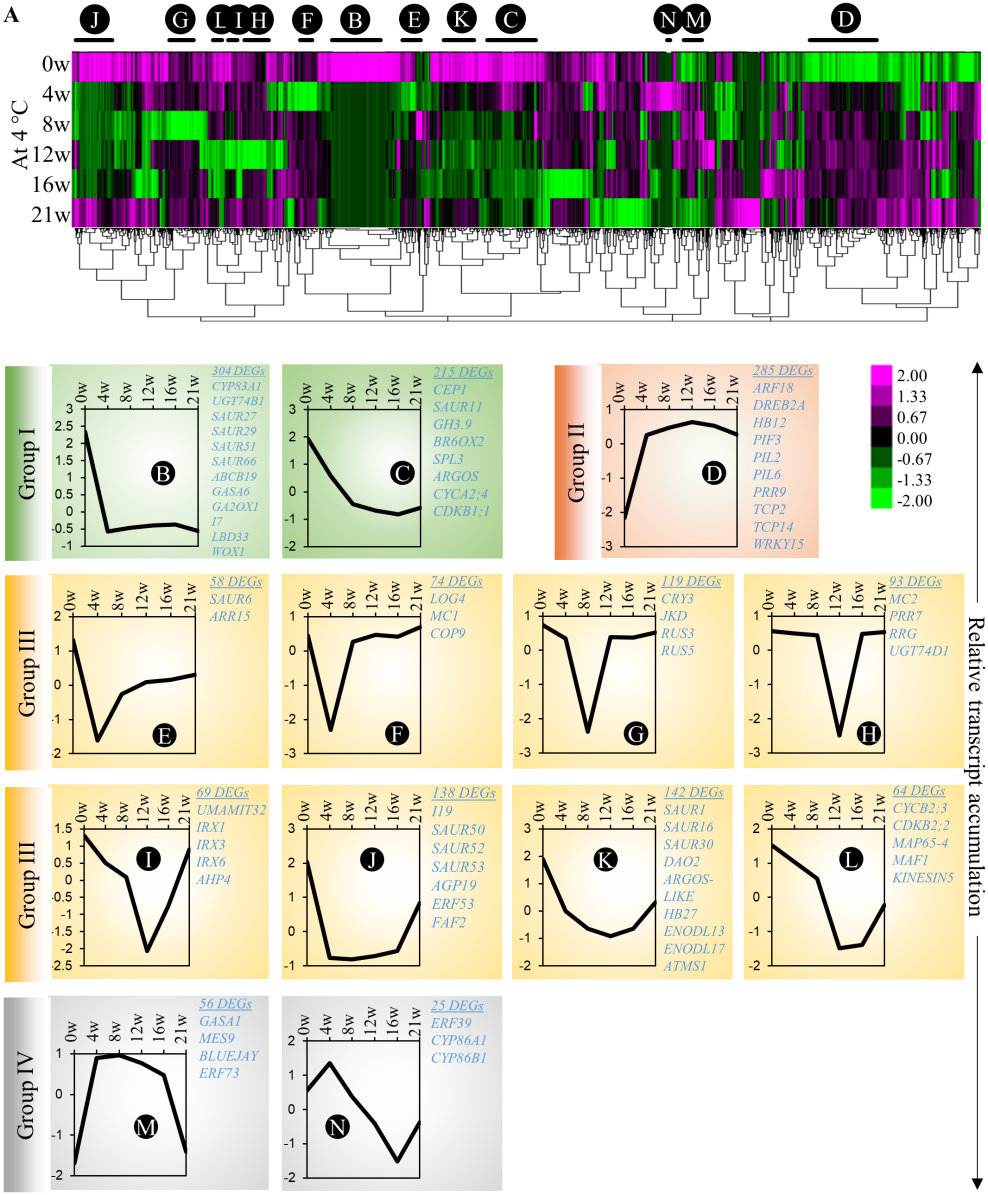
Co-expression clustering of the differentially expressed genes on *A alpina* stems. **(A)** The heat map shows the expression levels of selected genes (3540 genes) before cold exposure (0w), and 4w, 8w, 12w, 16w and 21w after cold exposure. For the heat map, genes were selected out of the total of 30510 genes based on the following criteria: log_2_(Fold-change) ≥ 2 and the difference between the maximum and the minimum value ≥ 3. The heat map was generated with Cluster 3.0 and was analysed with TreeView. Changes in the expression pattern are depicted as shown in the scale below the heat map. Green represents downregulation and magenta upregulation of expression levels. **(B–N)** The average normalized expression pattern of genes in selected co-expression clusters shown with black/red lines above the heat map. The expression levels were normalized with Cluster 3.0. The sub-clusters showing a similar expression pattern are highlighted in similar colours. The cluster sizes and co-expressed genes are indicated besides the co-expression plots. The heat map was divided into four major types (I - green, II - orange, III - yellow & IV - grey) based on the overall expression pattern. The GO enrichment of the clusters is presented in the **Supplementary Dataset 3**.

Our experimental design allows us to differentiate gene regulatory networks that participate in cold adaptation, adventitious primordia formation and emergence in cold (in cold-treated plants for 21 weeks) or in adventitious root emergence in response to the transition between cold and warm temperature (in cold-treated plants for 12 and 16 weeks). First, to identify molecular processes affected during cold-induced adventitious rooting in *A. alpina*, we investigated the transcriptomic changes in stems that take place during cold exposure (**Supplementary Dataset 2**). We constructed a heat map and looked for co-expression clusters that might explain the different developmental stages of adventitious root formation, including the induction and initiation of adventitious roots (**Figure 4**; **Supplementary Dataset 3**). To identify genes with strong changes in expression pattern, we selected genes that showed a more than four-fold change between the highest and the lowest expression values. Among all the *A. alpina* genes, 3540 genes fulfilled this criterion and were included for the co-expression analysis. The heat map was divided into 69 significantly enriched sub-clusters. We focused on 13 clusters that represented 46% of the total clustering (**Figure 4A**; **Supplementary Figure 4**; **Supplementary Dataset 4**). Among these, we identified clusters in which transcript accumulation of genes was downregulated during cold exposure (Group I; highlighted in *green*; Clusters B and C) and others in which gene expression was upregulated (Group II; highlighted in red; Cluster D). Genes in these groups may be involved in the induction phase of root primordia and play a negative or positive role during subsequent phases of root primordia formation. In Group I, we detected genes associated with auxin homeostasis (*GH3.9*, *CYP83A1* and *UGT74B1*), response (*IAA7*, *SPL3*, *SAUR11*, *SAUR27*, *SAUR29*, *SAUR51, SAUR66* and *SAUR67*) and transport (*ABCB19*); gibberellic acid degradation (*GA2OX1*), and signaling (*GASA6*); brassinosteroid degradation (*BR6OX2*) and meristem associated genes (*LBD33* and *WOX1*). Group II included genes involved in red/far-red light responses (*PIF3*, *PIL2* and *PIL6*) or associated with plant morphogenesis (*TCP2* and *TCP14*). While the genes in group I have not been specifically identified previously as transcriptionally regulated by cold, we cannot rule out the possibility that they may be associated with cold response independently of root primordia formation. The other groups exhibited a more specific pattern of expression at various time points during cold exposure. We detected clusters in which gene expression was transiently silenced at early (Group III; highlighted in yellow; Clusters E - L) or late stages of cold treatment (Group IV; highlighted in grey; Clusters M - N). Group III included genes involved in auxin response (*DAO2*, *SAUR1*, *SAUR6*, *SAUR16*, *SAUR50*, *SAUR52*, *SAUR53* and *IAA19*); dividing meristems (*ARGOS-LIKE*, *CYCB2;3*, *CDKB2;2*, *MAP65-4* and *KINESIN5*); cytokinin signaling (*ARR15*, *AHP4* and *LOG4*); and ethylene signaling (*ERF53*). Group IV includes genes associated with ethylene signaling (ERF39 and ERF73); suberin biosynthesis (*CYP86A1* and *CYP86B1*); gibberellic acid signaling and homoeostasis (*GASA1* and *MES9*) and stem cell regulator *BLUEJAY*. Transcript accumulation of genes in Group III might be involved in the induction/initiation phase of root primordium, whereas transcript accumulation of genes in Group IV in the emergence phase of root primordium. These data also suggest a downregulation of auxin response during the early phases of cold exposure as observed for the *DR5* auxin response in cold (**Supplementary Figure 5**; **Figure 3**). Accordingly, free auxin levels on stems were significantly reduced in plants after 4w in cold (**Supplementary Figure 5**). However, we observed a gradual (but not statistically significant) upregulation of endogenous IAA levels in stems of plants exposed to prolonged cold (**Supplementary Figure 5**).

**Figure 5:**
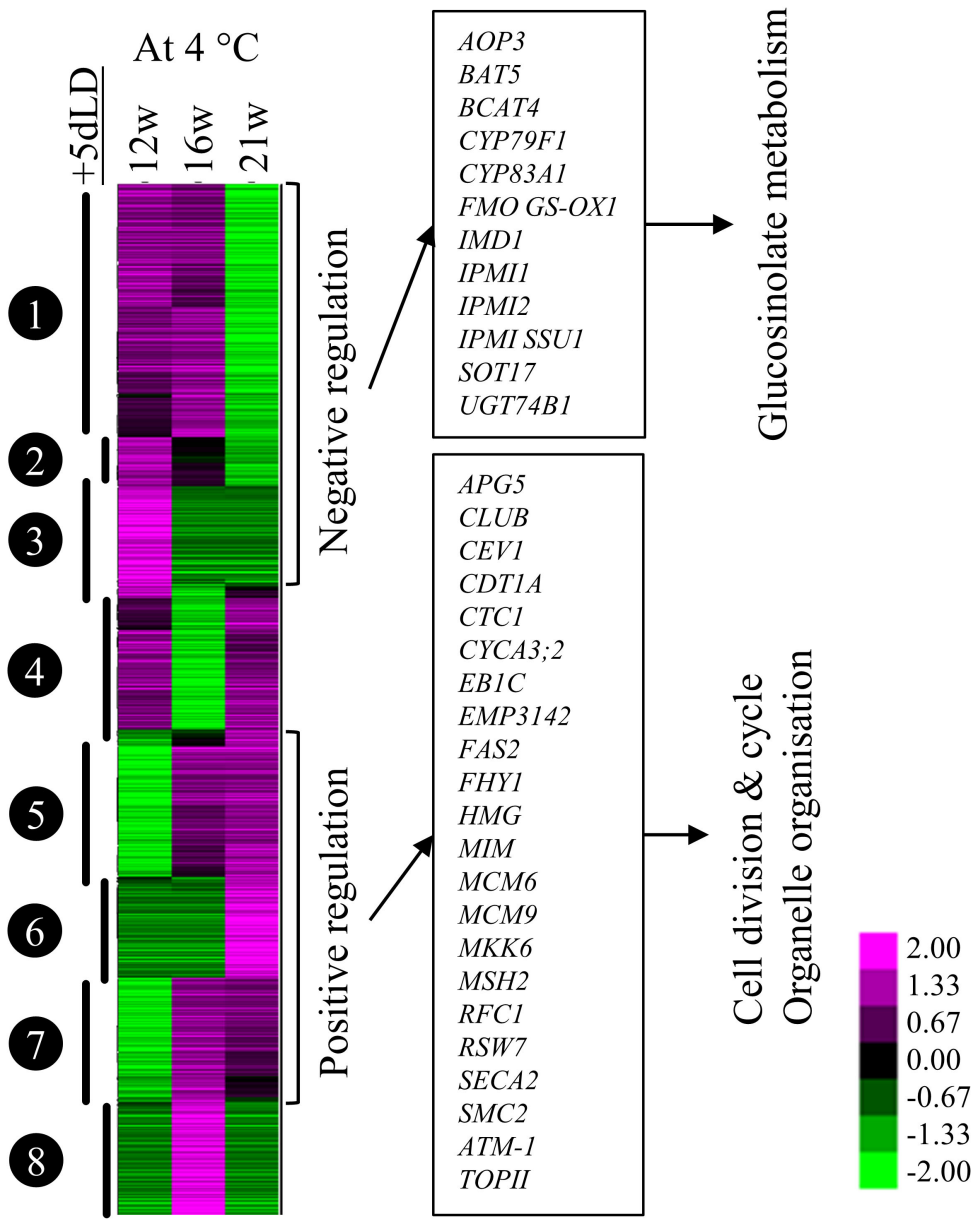
Co-expression clustering of the differentially expressed genes 5 days after cold exposure. Heatmap of the expression pattern of 3384 genes out of the 30690 identified in *A. alpina* with log_2_(Fold-change) ≥ 2 and the difference between the maximum and the minimum value ≥ 3. The heat map was generated with Cluster 3.0 and was analysed with TreeView. Changes in the expression pattern are depicted as shown in the scale below the heat map. Green represents downregulation and magenta represents upregulation of expression levels. The heat map shows the expression levels of selected genes in plants exposed to cold for 12w, 16w and 21w followed by 5 days in LD greenhouse.

To identify genes and processes involved in the emergence phase of adventitious roots, we investigated the transcriptomic changes in stems taking place after cold exposure. Stems of 21w cold-treated plants had fewer genes differentially regulated between samples harvested at the end (+0dLD) and after (+5dLD) cold treatment in comparison to the ones exposed to 12w or 16w of cold treatment (**Supplementary Figure 2B**; **Supplementary Table 1**). This result suggests that after 21w, the transcriptome response of *A. alpina* to cold treatment was saturated, as was the number of plants producing cold-dependent adventitious roots. We performed a co-expression analysis of the genes detected between stems harvested at the end and after cold treatment (**Figure 5**). We found that the accumulation of transcripts involved in glucosinolate metabolism (including *CYP83A1*, *CYP79F1*, *UGT74B1*, *SOT17*, *IMD1*, *IPMI1* and *IPMI2*) was reduced after 21 weeks of cold treatment, whereas it was comparatively higher after 12 and 16 weeks of cold treatment. Inversely, transcript accumulation of genes participating in cell cycle, cell division and organelle reorganization (among them *ATCDC48B*, *CYCA3;2*, *EB1C*, *MCM2*, *MCM6*, *MCM9*, *MKK6*, *SMC2* and *TOPII*) were significantly increased in stems of 21w cold treated plants compared to the stems of plants cold-treated for 12 and 16 weeks (**Figure 5**). Homologs of ethylene biosynthesis genes *ACO3* and *ACS6*, ethylene signaling genes *ERF6* and *ERF104* and the auxin signaling gene *IAA19* were also upregulated after extended cold exposure (**Supplementary Dataset 2**).

### 3.4 Adventitious rooting in *A. alpina* correlates with the differential regulation of hormone- associated genes during and after cold treatment

**Figure 6:**
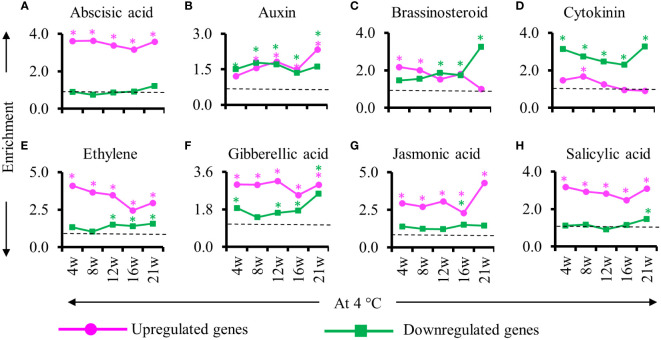
Enrichment of genes responsive to hormones during cold exposure. **(A–H)** Enriched hormone responsive genes in stems at the end of cold (4, 8, 12, 16, 21 weeks at 4°C) relative to before cold exposure. **(A)** Abscisic acid, **(B)** auxin, **(C)** brassinosteroid, **(D)** cytokinin, **(E)** ethylene, **(F)** gibberellic acid, **(G)** jasmonic acid and **(H)** salicylic acid responsive genes are represented. The upregulated (magenta) and downregulated (green) genes are represented. The enrichment value and enrichment test were calculated as mentioned in the Materials and Methods. Stars indicates *P* < 0.05.

As hormones are instrumental in the regulation of adventitious roots, we examined the enrichment of phytohormone-related genes among the DEGs identified during cold treatment during the formation of adventitious root primordia (**Figure 6**). We found that abscisic acid, auxin, ethylene, gibberellic acid, jasmonic acid and salicylic acid responsive genes were enriched in the upregulated DEGs detected throughout cold exposure except for the auxin response gene at 4 weeks into cold. Among the downregulated genes, we detected an enrichment of auxin, Cytokinin and gibberellic acid-responsive genes (**Figure 6**). Notably, brassinosteroid response gene were enriched only in upregulated genes at the beginning of the cold period (4 and 8 weeks) and in down regulated at the second part of the cold exposure (12, 16 and 21 weeks) suggesting an opposite role of this hormone in adventitious root primordia initiation/formation and maturation/emergence in cold (**Figure 6C**). Auxin-responsive genes showed similar enrichment among the upregulated and downregulated genes (**Figure 6B**). However, at the end of 21w of cold exposure the number of auxin responsive genes in the upregulated gene was twice as high compared to the other time points (**Figure 6B**). Concurrently, jasmonic acid responsive genes were overrepresented among the upregulated gene and brassinosteroid responsive genes in the downregulated genes. These results point to changes in auxin, jasmonic acid, brassinostreroid and cytokinin response specifically at 21w at 4°C.

**Figure 7:**
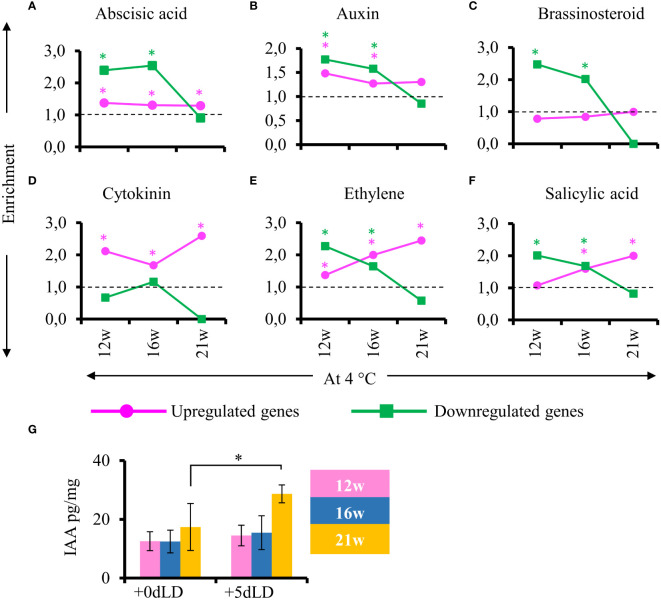
Enrichment of genes responsive to hormones after cold exposure. **(A–F)** Enriched hormone responsive genes in stems 5 days after transfer to LD greenhouse (+5dLD) conditions relative to the end of cold (12, 16, 21 weeks at 4°C). **(A)** Abscisic acid, **(B)** auxin, **(C)** brassinosteroid, **(D)** cytokinin, **(E)** ethylene and **(F)** salicylic acid responsive genes are shown here. The upregulated genes are represented in magenta and downregulated genes in green. The enrichment value and enrichment test were calculated as mentioned in the Materials and Methods. Stars indicates *P* < 0.05. **(G)** Quantification of auxin (IAA pg/mg) in the stems at the end of 12w, 16w and 21w of cold (4°C), and 5 days after transfer to LD conditions (+5dLD). The mean and the standard deviation represent three biological replicates. A one-way analysis of variance (ANOVA) followed by Tukey’s multiple comparison *post hoc* test with Bonferroni correction was used to determine significantly different samples (p < 0.05) as presented in **Supplementary Table 3**. Stars indicate P < 0.05.

To determine the regulation of hormone responsiveness after cold exposure, we looked for hormone responsive genes in stem samples harvested from plants at the end of cold treatment and after plants had been returned to greenhouse conditions. No gibberellic acid and jasmonic acid responsive genes were identified among the DEGs. Interestingly, genes responsive to abscisic acid, auxin, brassinosteroid, ethylene and salicylic acid were enriched among the down regulated genes in cold-treated plant for 12 and 16 weeks but not 21 weeks (**Figure 7**). Specifically, in stems of plants cold treated for 21w, we observed an exceptionally low enrichment of DEGs related to hormones compared to the ones in plants cold treated for 12w or 16w. This pattern was also observed for the auxin responsive genes. Nevertheless, endogenous IAA levels in stems increased in plants transferred to greenhouse conditions after 21 weeks of cold treatment (**Figure 7G**). This result suggests that hormones are also important for the later stages of adventitious rooting and explains the lack of hormone responsive DEGs in stems of 21 weeks cold treated plants that develop adventitious roots already during cold treatment. The hormone response for adventitious rooting is already primed in cold by 21 weeks.

## 4 Discussion

**Figure 8:**
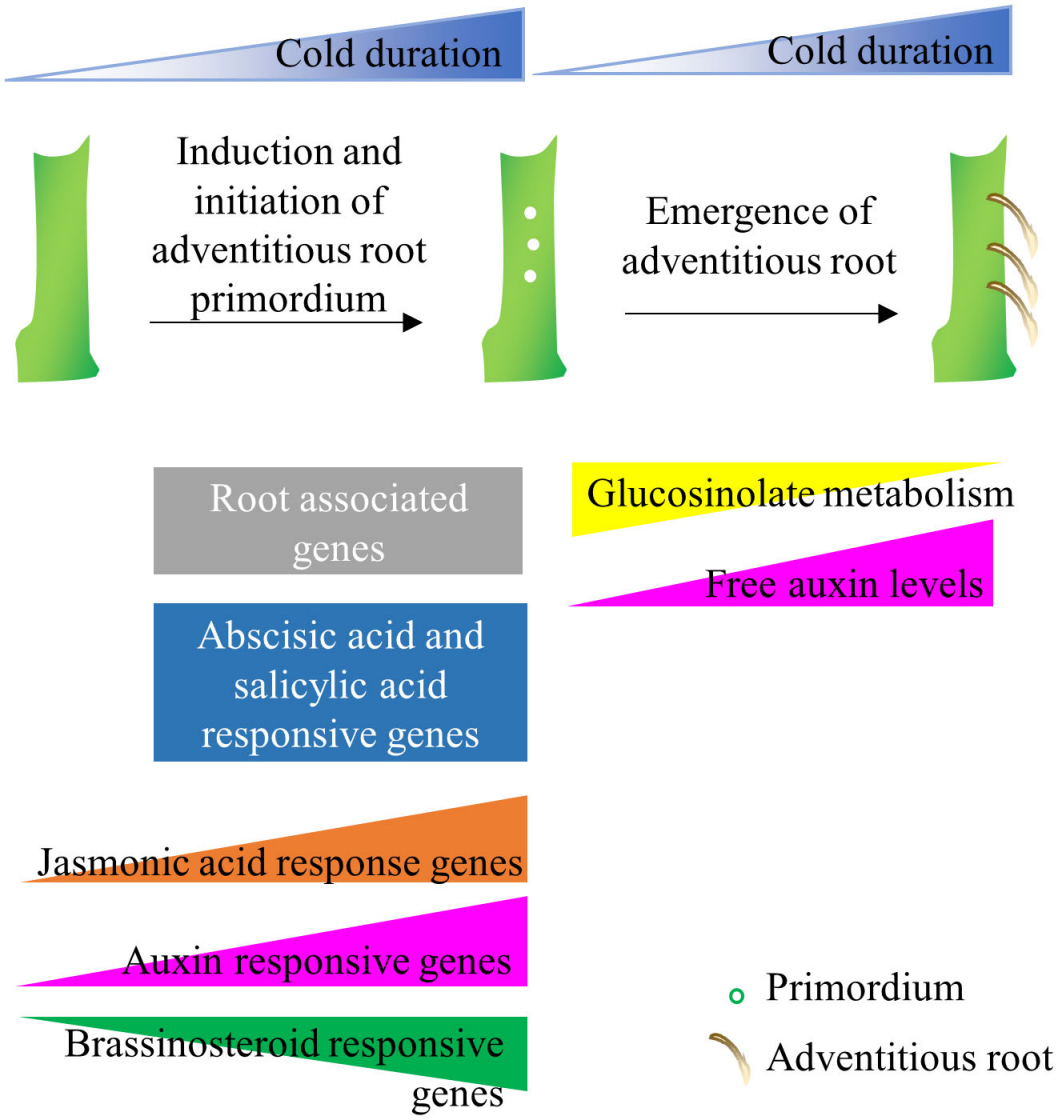
Model of adventitious root formation in response to long-term cold. Adventitious root primordia are induced during cold exposure and emerge after plants experience growth promoting conditions. The initiation of adventitious root primordia on stems correlates with the upregulation of root-associated genes, whereas the emergence of adventitious roots associate with hormone responsive genes, glucosinolate metabolism genes and endogenous IAA levels.

Alpine environments are often characterized by short and unpredictable growing seasons, which put additional constraints on the survival of alpine species (Körner, 1995). Thus often, alpine plants use both sexual and asexual means of reproduction to distribute risk and survive under severe environmental conditions (Körner, 2003). Ecological studies on alpine flora have demonstrated that the flowering process in alpine species has been modified so that plants will reproduce rapidly in short growth seasons (Diggle, 1997; Meloche and Diggle, 2001). Specifically, alpine plants induce flowering in response to prolonged cold but also flowering meristems initiate and grow during cold exposure several months in advance to the flowering season (Diggle, 1997; Meloche and Diggle, 2001). The arctic-alpine perennial *A. alpina* follows a similar reproductive strategy, as demonstrated in controlled environment experiments by exposing plants to different lengths of cold treatment but also observed in natural *A. alpina* populations (Wang et al., 2009; Toräng et al., 2015; Lazaro et al., 2018). Here, we explored the role of prolonged cold treatment on adventitious rooting. As shown previously for flowering, cold exposure has a quantitative effect on adventitious rooting and adventitious roots are induced and initiated during cold exposure (**Figure 8**). Long-term cold being a trigger for the induction and initiation of flowering and adventitious root meristem formation suggests that alpine species may use the same environmental cue to initiate both processes simultaneously and ensure balancing of the trade-off between the reproductive strategies (Wang et al., 2009; Lazaro et al., 2018). These results may explain how alpine species are able to switch rapidly between sexual or asexual reproductive strategies. Alpine species, such as *Geum reptans*, *Epilobium* and *Polygonum viviparum* are also able to employ clonal propagation and sexual reproduction simultaneously (reviewed in Körner, 2003). This hypothesis, however, is not in line with possible trade-offs between sexual and asexual reproduction that are usually displayed under limiting resources (Gardner and Mangel, 1999). Evidently, to minimize the trade-offs, most plant species that practice both sexual and asexual reproduction stagger the alternative reproductive strategies (Hossaertmckey et al., 1992; Cheplick, 1995; Gardner and Mangel, 1999; Méndez, 1999; Thompson and Eckert, 2004; Van Drunen and Dorken, 2012).

The relationship of flowering and adventitious rooting has been addressed in non-alpine species using rooting experiments on cuttings of flowering time mutants or transgenic lines. Pea cuttings harvested from plants after the transition to flowering fail to produce adventitious roots, demonstrating a negative association between flowering and adventitious rooting (Rasmussen et al., 2015). Here, we observe the co-occurrence of flowering and adventitious rooting in the Pajares accession. In addition, *the A. alpina* accession Wca that does not require cold exposure to flower also produces flowers and adventitious roots simultaneously (Mishra et al., 2020). These results suggest the absence of a trade-off between vegetative and sexual propagation and of the shared regulation of both processes in *A. alpina*. MicroRNA 156 (*miR156*) and its targets, that belong to the SQUAMOSA BINDING PROTEIN LIKE family, are involved in the regulation of flowering through the age pathway and are also implicated in the regulation of other traits, including lateral root and adventitious root formation (Wu and Poethig, 2006; Yu et al., 2015; Zheng et al., 2019). The role of *miR156* on adventitious rooting is evident in plants overexpressing *miR156*, such as in maize the *Congrass 1* mutant, or in tomato and tobacco transgenic lines (Chuck et al., 2007; Zhang et al., 2011; Feng et al., 2016). Plants constitutively expressing *miR156*, flower late and exhibit dense adventitious roots in their stems, demonstrating again a negative association of flowering with adventitious rooting (Chuck et al., 2007; Zhang et al., 2011; Feng et al., 2016). miR156 represses the juvenile to adult phase transition, in which plants become competent to flowering (Wu and Poethig, 2006). Thus, apices of young (non-competent) seedlings have a high accumulation of *miR156*. Similarly, cuttings from juvenile plant parts have a higher rooting ability and higher accumulation of *miR156* compared to adult cuttings (Xu et al., 2017). Overall, *miR156* has an opposite role in these processes, as it represses flowering and promotes adventitious rooting. *miR156* and *AaSPL15* transgenic lines in *A. alpina* have been used to study the role of these age-related factors on flowering but their adventitious rooting phenotype has not been investigated (Bergonzi et al., 2013; Hyun et al., 2019). In this study, we find the transcript accumulation of *SPL3*, an auxin responsive gene, downregulated during cold exposure as competence to adventitious rooting increases. The spatial pattern of adventitious rooting which we observed (at lower and at higher internodes) cannot be explained by age-related differences between internodes, as higher internodes are chronologically but not physiologically younger than the internodes beneath (Rasmussen et al., 2015). Further studies addressing the role of age in vegetative propagation through adventitious rooting are required.

The duration of cold exposure influences the speed of the emergence of adventitious roots after plants return to glasshouse conditions. A similar effect of cold exposure was observed previously in *A. alpina* in the case of the emergence of flower buds initiated during cold (Lazaro et al., 2018). Abscisic acid and salicylic acid together engage in cold stress tolerance in rice, maize, and wheat (Pál et al., 2011; Zhao et al., 2015; Wang et al., 2018). In this study, abscisic acid and salicylic acid signaling were upregulated consistently throughout cold exposure indicative of an activated stress response in the *A. alpina* stem. In our data, the role of auxin and other phytohormones in the distinct phases of adventitious root formation during cold exposure is not noticeably clear. Nevertheless, we detected several candidates related to phytohormone signaling that might participate at the various stages of adventitious rooting. Previous studies in *Arabidopsis* demonstrated that cold affects auxin transport but not signaling (Shibasaki et al., 2009). However, in *A. alpina*, prolonged cold exposure was found to decrease auxin transport capacity in the stem, IAA levels and DR5 auxin response (Vayssières et al., 2020). Conversely, we found several auxin signaling genes (*ARF*, *Aux/IAA* and *SAUR* family) upregulated and enriched during 21-week cold exposure suggestive of selective activation of auxin signaling. Keeping up with the role of cytokinin as a negative regulator of adventitious rooting, we detected cytokinin signaling genes to be downregulated throughout cold exposure (Lakehal and Bellini, 2018; Lakehal et al., 2020). During the emergence phase of adventitious roots, we detected an upregulation of genes involved in ethylene biosynthesis and signaling. Ethylene signaling is required for the death of selective epidermal cells located above the adventitious root primordia, suggesting that the role of ethylene on adventitious root emergence is conserved in *A. alpina* (Mergemann and Sauter, 2000; Steffens and Sauter, 2009a, 2009b).

While glucosinolates were not typically associated with adventitious root formation, we also found an association between the downregulation of glucosinolate metabolism and the increase in competence to form adventitious root in long-term cold. Glucosinolates are sulfur-containing secondary metabolites found in plants of the order Brassicales (Blažević et al., 2020). Specifically, the glucosinolates with indole as their side chain are linked to auxin homeostasis. Interruption of glucosinolate biosynthesis causes phenotypes that resemble high auxin levels (Boerjan et al., 1995; Mikkelsen et al., 2004). In this transcriptomic study, we found the expression of a cytochrome P450 *CYP83A1* was downregulated after 21 weeks of vernalization. A homolog of *CYP83A1*, *CYP83B1* also plays role in indole glucosinolate biosynthesis and adventitious root formation (Delarue et al., 1998; Barlier et al., 2000; Bak and Feyereisen, 2001; Bak et al., 2001; Pacurar et al., 2014). Likewise, the UDP-glucose:thiohydroximate S-glucosyltransferase, *UGT74B1*, whose abundance decreases as adventitious rooting increased, also regulates balance between glucosinolate and auxin biosynthesis (Grubb et al., 2004). Similarly, *CYP79F1*, another gene involved in glucosinolate metabolism, was also downregulated in 21-week cold-treated main stem (Falasca et al., 2010). Moreover, the inhibitory role of long-term cold exposure on indole glucosinolates content was revealed in vernalized radish plants (Nugroho et al., 2021). Our study suggests that the inhibition by long-term cold of specific glucosinolate-derived compounds in the rooting environment may influence hormone signaling pathways, especially auxin, potentially affecting the growth of adventitious roots (**Figure 8**). However, further studies would be required to identify whether glucosinolates directly regulate adventitious rooting during cold exposure or their impact is indirect through auxin metabolism. In conclusion, our work on *A. alpina* can give insights into the regulators of adventitious rooting and optimize horticultural practices which rely on cold storage to promote adventitious root formation in stem cuttings.

## Data availability statement

The data presented in the study are deposited in the GEO repository, accession number GSE176054.

## Author contributions

PM: Conceptualization, Data curation, Formal analysis, Investigation, Writing – original draft. AR: Investigation, Writing – review & editing. KL: Investigation, Writing – review & editing. MA: Conceptualization, Data curation, Funding acquisition, Investigation, Project administration, Supervision, Writing – original draft. AV: Conceptualization, Data curation, Investigation, Supervision, Writing – review & editing.

## Supplementary material

Supplementary Figure 1 Physiological analysis of *A. alpina* without cold exposure and in response to different durations of cold. Percentage of flowering plants **(A)** and total leaf number at flowering **(B)** in plants without cold exposure (0w) and exposed to different durations (4w, 8w, 12w, 16w and 21w) at 4°C followed by 4 weeks in long days (+4wLD). A one-way analysis of variance (ANOVA) followed by Tukey’s multiple comparison *post hoc*-test with Bonferroni correction showed no statistically significant value. **(C)** Diagrammatic representation of the experimental design of the physiological analysis in *A. alpina* without cold exposure. Plants were grown for eight weeks in a LD greenhouse and subsequently maintained in LD for 5 additional weeks. (**D)** Analysis of the position of adventitious root formation along the main stem in a set of *A. alpina* plants grown 13 weeks in a LD greenhouse corresponding to eight weeks plants scored for additional 5 weeks. Each column represents a single plant. The percentage of adventitious root in each internode along the main stem is indicated in orange on the right side of the graph. Internodes are numbered from the bottom to the top of the plant. Percentage of Pajares plants with adventitious roots on the main stem **(E)** and the axillary branches **(F)** 5 weeks in long days (+5wLD).

Supplementary Figure 2 Overview of the transcriptomic composition during cold exposure. Venn diagram depicting the number of genes regulated during **(A)** extended cold (4°C) relative to before cold exposure and **(B)** 5 days after transfer to LD greenhouse conditions relative to end of cold treatment (12, 16 or 21 weeks at 4°C). The number of genes upregulated (magenta) and downregulated (green) are represented for each set, and genes that are similarly regulated between the increasing duration of cold are shown at the intersection (**Supplementary Dataset 2**). **(C)** The tree shows the distribution of stem transcriptome collected at different time-points. The cluster is divided into two sub-clusters representing the samples collected during cold (4°C) (highlighted in blue) and samples collected 5 days after transfer to LD greenhouse (highlighted in yellow).

Supplementary Figure 3 Relative transcript accumulation of the *GUS* transcript with respect to the housekeeping gene *AaPP2A* in the cold-dependent rooting zone of *pDR5::GUS* Pajares plants before cold exposure and during cold exposure (4°C) for 4w, 8w, 12w, 16w and 21w.

Supplementary Figure 4 Co-expression clustering of the differentially expressed genes during extended cold exposure. The average normalized expression pattern of genes in each cluster is plotted. The expression levels are normalized with Cluster 3.0. The number of each cluster follows the tables in **Supplementary Dataset 4**.

Supplementary Figure 5 Quantification of auxin (IAA pg/mg) in the stems before cold treatment (0w), and at the end of 4, 8, 12, 16 and 21 weeks at 4°C. The mean and the standard deviation represent three biological replicates. A one-way analysis of variance (ANOVA) followed by Tukey’s multiple comparison *post hoc*-test with Bonferroni correction was used to determine significantly different samples (p < 0.05) as presented in **Supplementary Table 3**.
