# Supplementary material for: Adventitious rooting in response to long-term cold: a possible mechanism of clonal growth in alpine perennials
Source: Front Plant Sci. 2024 Apr 17;15:1352830. doi: 10.3389/fpls.2024.1352830 (PMC11062184; doi:10.3389/fpls.2024.1352830)
Supplement: Supplementary Figure 1 — Physiological analysis of A. alpina without cold exposure and in response to different durations of cold. Percentage of flowering plants (A) and total leaf number at flowering (B) in plants without cold exposure (0w) and exposed to different durations (4w, 8w, 12w, 16w and 21w) at 4°C followed by 4 weeks in long days (+4wLD). A one-way analysis of variance (ANOVA) followed by Tukey’s multiple comparison post hoc-test with Bonferroni correction showed no statistically significant value. (C) Diagrammatic representation of the experimental design of the physiological analysis in A. alpina without cold exposure. Plants were grown for eight weeks in a LD greenhouse and subsequently maintained in LD for 5 additional weeks. (D) Analysis of the position of adventitious root formation along the main stem in a set of A. alpina plants grown 13 weeks in a LD greenhouse corresponding to eight weeks plants scored for additional 5 weeks. Each column represents a single plant. The percentage of adventitious root in each internode along the main stem is indicated in orange on the right side of the graph. Internodes are numbered from the bottom to the top of the plant. Percentage of Pajares plants with adventitious roots on the main stem (E) and the axillary branches (F) 5 weeks in long days (+5wLD). [file DataSheet_1.zip › Supplementary_Figures.pdf]

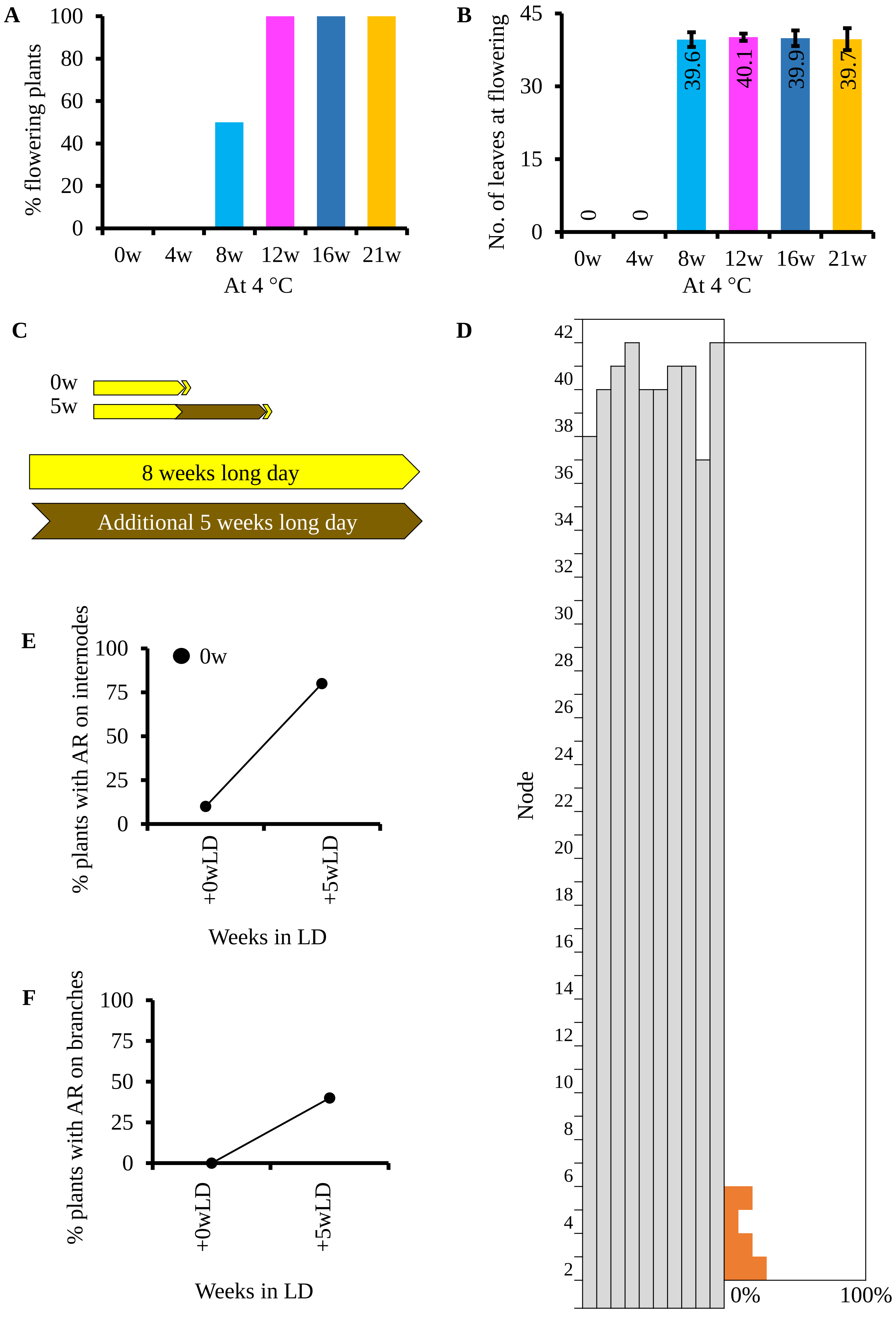

Supplementary Figure 1.

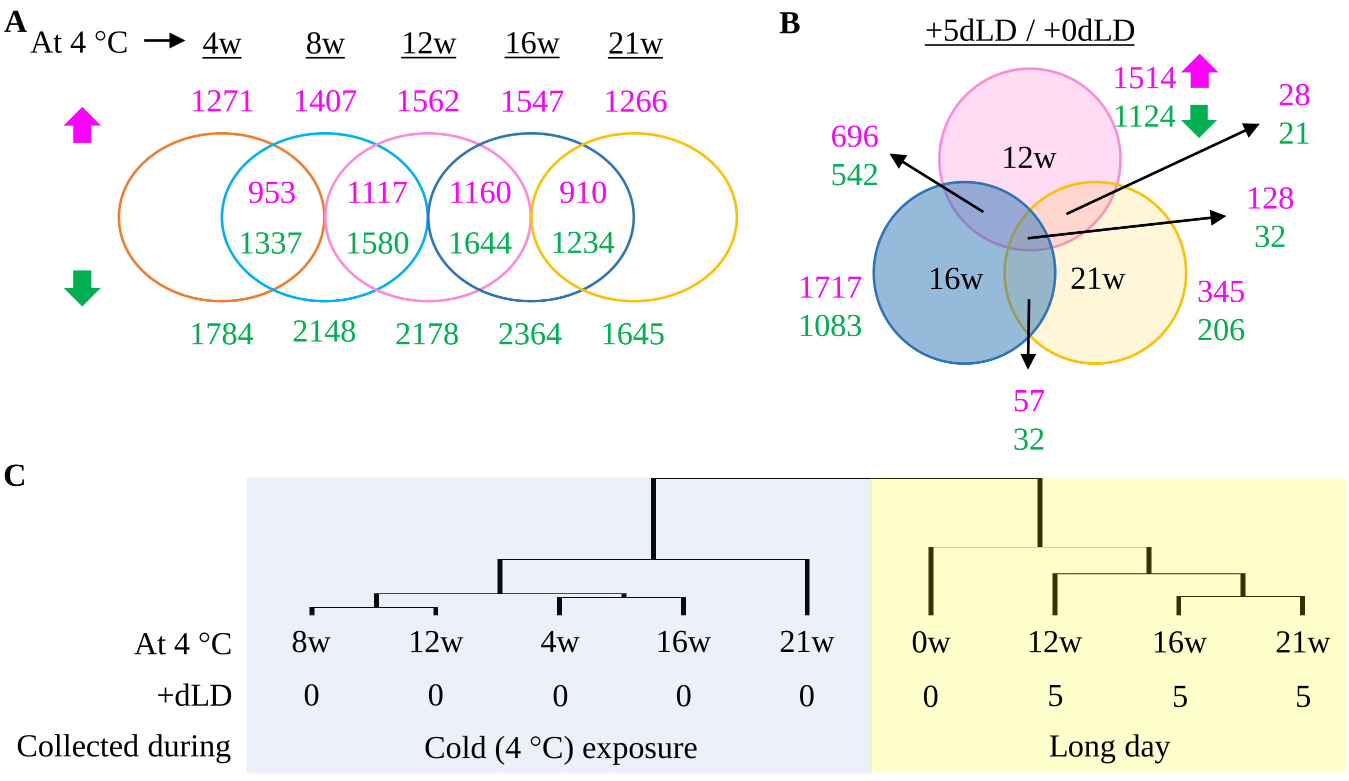

Supplementary Figure 2.

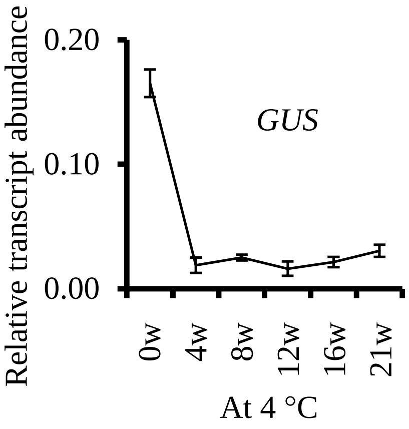

Supplementary Figure 3.

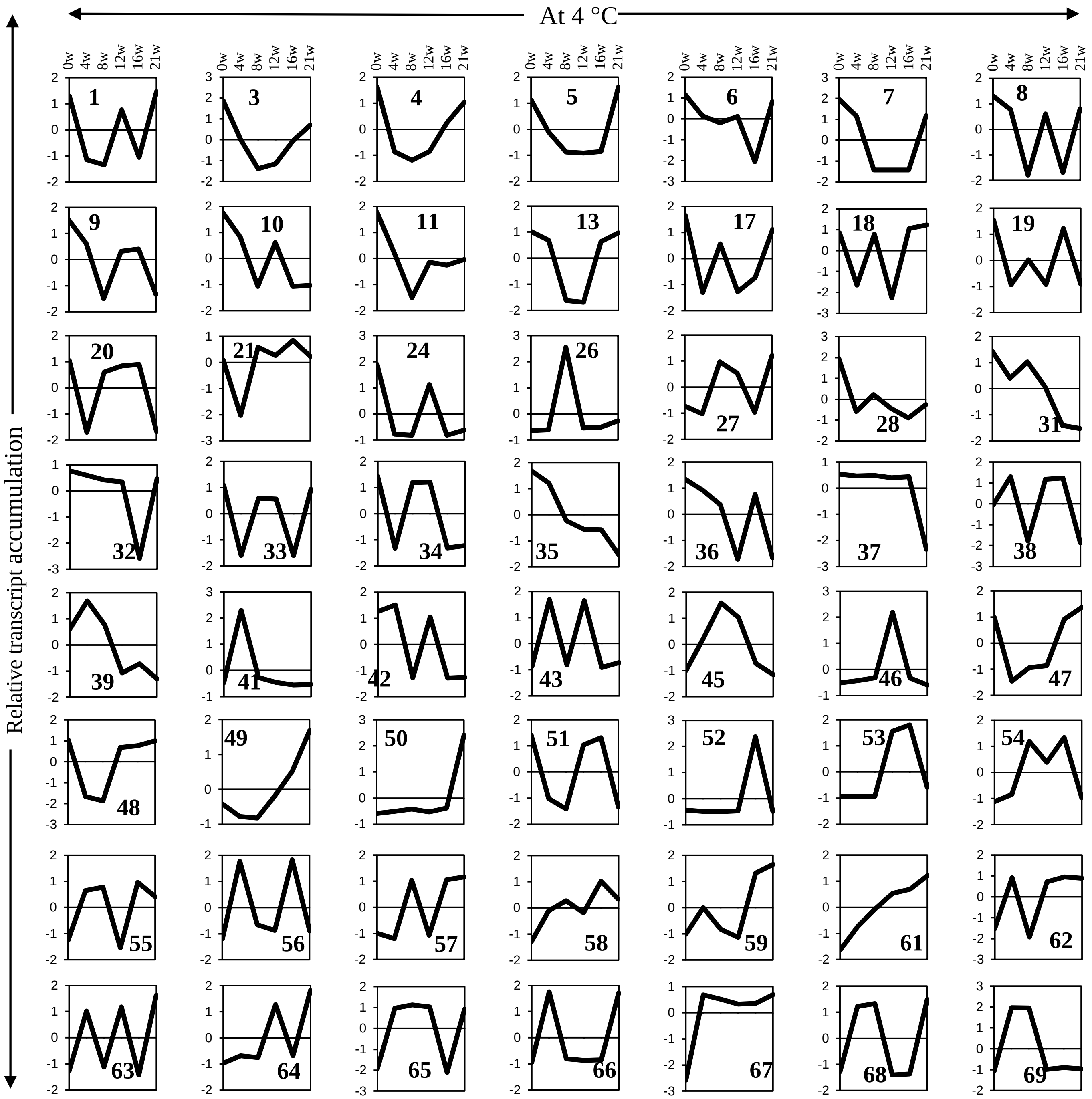

Supplementary Figure 4.

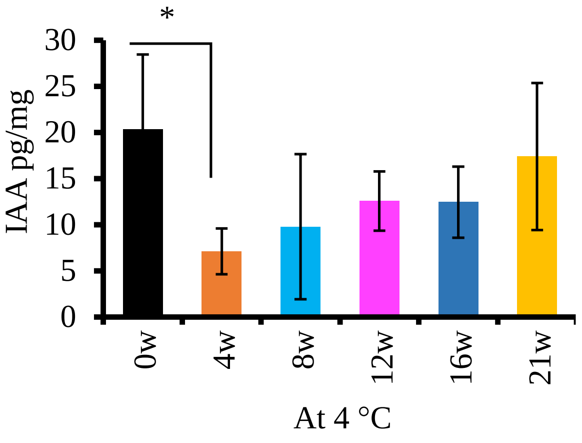

Supplementary Figure 5.
